# Comparative Review of Superior Capsule Reconstruction and Reverse Shoulder Arthroplasty for Irreparable Rotator Cuff Tear

**DOI:** 10.7759/cureus.101857

**Published:** 2026-01-19

**Authors:** Yusra Soorya, Yousef N Mosleh, Razan F J Qamar, Adegbenro O Fakoya

**Affiliations:** 1 Anatomy, School of Medicine, Louisiana State University Health Sciences Center, Shreveport, USA; 2 Cellular Biology and Anatomy, Louisiana State University Health Sciences Center, Shreveport, USA

**Keywords:** reverse shoulder arthroplasty, rotator cuff tear, shoulder graft rejection, shoulder joint stability, superior capsule reconstruction

## Abstract

Rotator cuff tears are a prevalent cause of shoulder dysfunction, particularly in aging populations, with conservative treatments often proving ineffective for massive or irreparable tears. When non-surgical options fail, superior capsule reconstruction (SCR) and reverse shoulder arthroplasty (RSA) are two primary surgical techniques utilized to restore shoulder function. SCR involves reconstructing the superior capsule using a graft to stabilize the glenohumeral joint and maintain native biomechanics. Conversely, RSA replaces the joint with a prosthetic system, altering shoulder mechanics to improve mobility and pain relief. While SCR preserves natural anatomy and is associated with positive functional outcomes, it presents challenges, including graft integrity issues and a high failure rate. RSA offers reliable pain relief and functional improvements, particularly in elderly patients, but is associated with complications such as implant loosening and scapular notching. This review explores the indications, surgical techniques, clinical outcomes, limitations, and cost-effectiveness of SCR and RSA for the treatment of irreparable rotator cuff tears. Studies indicate that while both procedures improve function and reduce pain, RSA generally provides more consistent long-term outcomes. In contrast, SCR may be preferable for younger, active patients seeking to delay arthroplasty. Additionally, cost considerations may influence surgical decisions: SCR is less invasive but may require revision surgery, whereas RSA entails higher initial costs but offers greater durability. By comparing these techniques, this review aims to aid in treatment selection based on patient-specific factors, such as age, activity level, and surgical risk.

## Introduction and background

**Figure 1:**
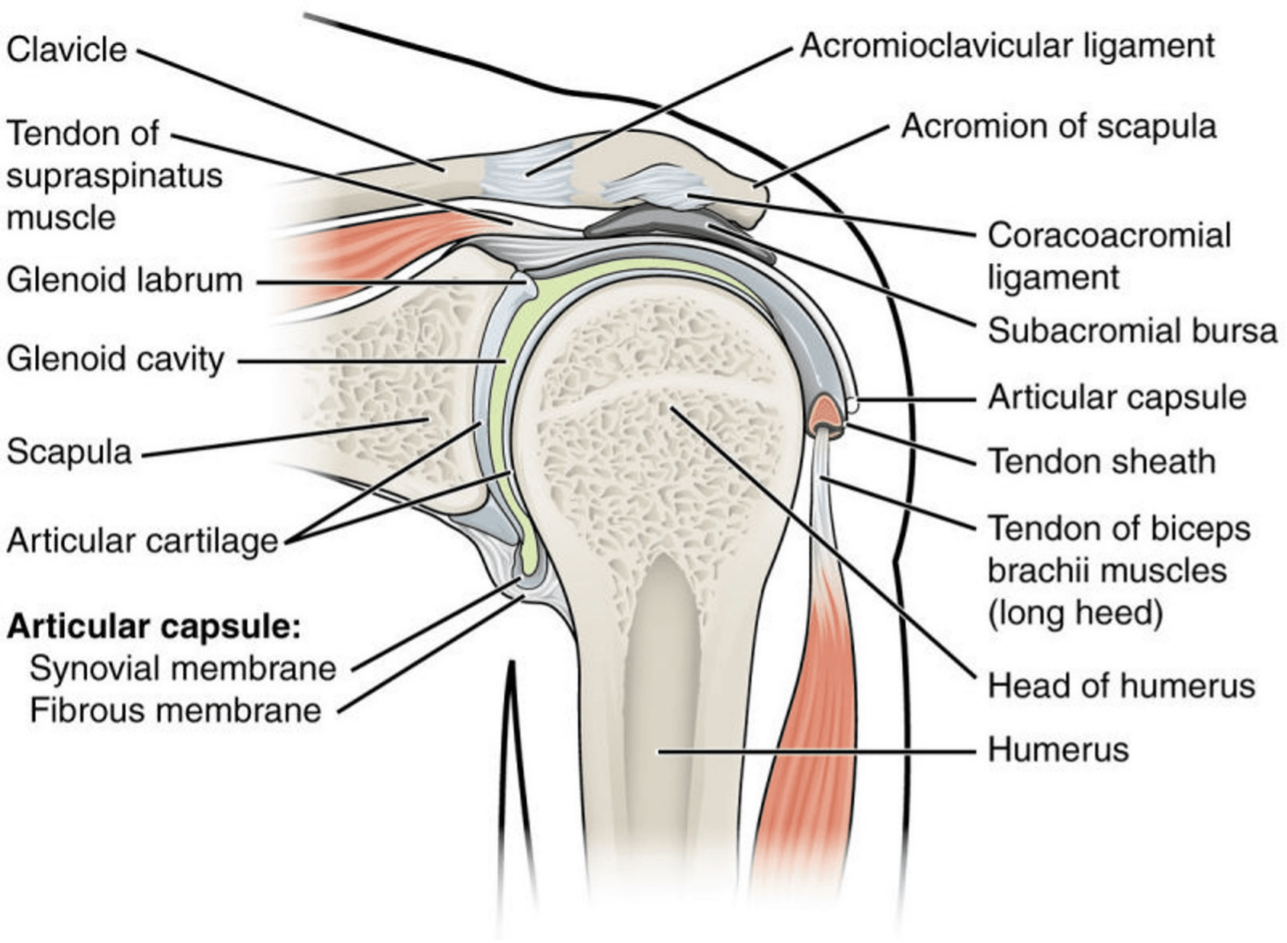
Shoulder joint anatomy. Anatomy of the shoulder joint, showing the supraspinatus muscle and tendon. Source: Figure adapted from Varacallo et al. [1] under the terms of the Creative Commons Attribution-NonCommercial-NoDerivatives 4.0 International (CC BY-NC-ND 4.0).

The shoulder joint, also known as the glenohumeral joint, is one of the most versatile joints in the body, allowing movements such as flexion, extension, abduction, and rotation. However, with this high mobility, instability can occur, and the rotator cuff plays a role in this process. The rotator cuff is made up of four muscles: the supraspinatus, infraspinatus, teres minor, and subscapularis [1]. These muscles work together to create a cuff that keeps the humerus (upper arm bone) in place at the glenoid cavity of the scapula (shoulder blade) and allows for a wide range of movement of the arm (Figure 1). The rotator cuff works with the deltoid muscle to lift and rotate the arm while maintaining glenohumeral joint stability.

**Figure 2:**
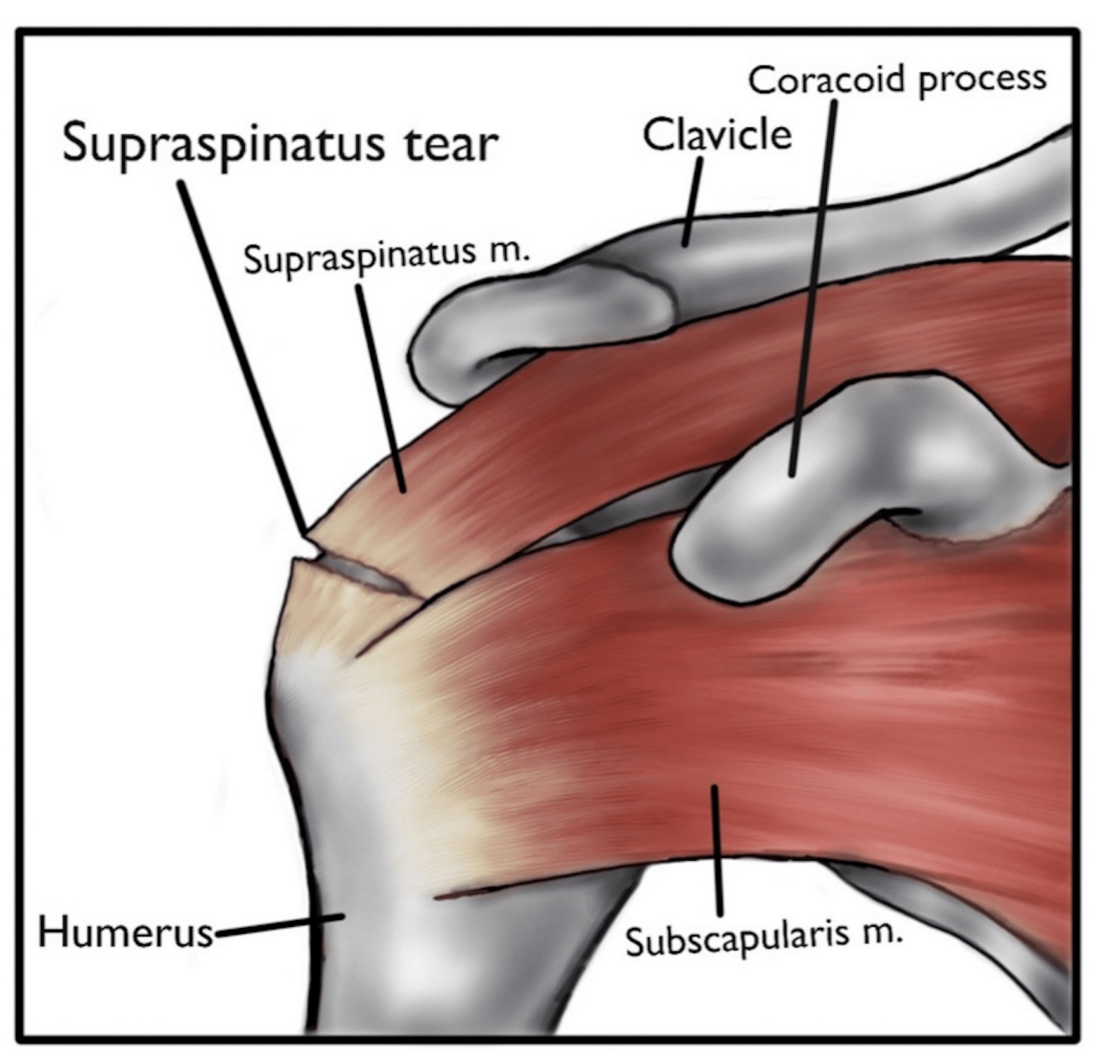
Rotator cuff tear. Rotator cuff tear involving the supraspinatus muscle. m: muscle. Source: Created by the authors.

A rotator cuff tear (RCT) is an injury to one or more of these muscle tendons (with the supraspinatus tendon being the most common), minimizing the functionality and restricting the movement of the arm (Figure 2). This disrupts the mechanical balance required to maintain joint stability and alters the biomechanics of the glenohumeral joint. RCTs can lead to a destructive cascade that results in secondary injury, superior migration of the humeral head, and progressive degeneration of joint cartilage and intact tendons [2]. This injury leads to decreased strength and range of motion, resulting in pain, weakness, and instability. With age and use, the likelihood of a rotator cuff injury increases. A population-based study of 683 participants (1,366 shoulders) found that 20.7% had an RCT, including 16.9% of subjects without symptoms. The study showed RCTs to be associated with increased age, dominant arm, males, history of trauma, and subjects who do heavy labor [3].

Initial treatments for RCT typically include conservative measures such as rest, physical therapy, and bracing. If conservative measures fail, patients will need to undergo surgical repair and reattachment of the torn tendon. However, suppose there is a recurrence of RCTs or even a massive RCT. In that case, it can cause irreparable damage to the surrounding tissue, causing tendon scarring and retraction, muscle atrophy, and fatty infiltration [4]. Furthermore, for larger, irreparable tears, reattachment will not suffice, and more aggressive measures may be considered, such as joint reconstruction [5]. Two reconstruction procedures discussed in this paper are the superior capsule reconstruction (SCR) and the reverse shoulder arthroplasty (RSA).

The superior capsule is a fibrous structure that is attached to the upper part of the glenoid cavity, and it stabilizes the humeral head from displacing superiorly in the glenohumeral joint. This capsule is shown to be associated with damage to the rotator cuff, specifically the supraspinatus and infraspinatus (Figure 2). The SCR is a surgical procedure that allows reconstruction of the capsule using various grafts (fascia lata autografts, dermal autografts, long head of bicep autografts, and porcine dermis xenografts). A systematic review and meta-analysis by Werthel et al. examined 18 studies on the SCR and found improvements in the range of motion and pain. The complication rate of this surgery was 5.6%, with surgical site infection, bicep pain, donor site complications, anchor pullouts, and acute immune rejection of the xenograft being the most common [6].

On the other hand, the RSA is currently the most commonly performed method for shoulder reconstruction. The RSA is a prosthetic reconstructive surgery that rearranges the natural glenohumeral joint orientation by shifting the center of rotation medially and inferiorly. This is done by placing the “ball” portion of the joint on the glenoid and the “socket” portion on the humerus, therefore reversing the ball-and-socket joint. This allows the deltoid muscle to overcompensate for the rotator cuff deficiency. A systematic review and meta-analysis by Sevivas et al. on RSA showed that it significantly improved pain-related function and mobility from preoperative to postoperative status in patients with massive RCTs. However, the complication rate was as high as 20%, and the revision rate was one in 12 cases [7], with a retear of the rotator cuff being the most common complication [8]. Ultimately, SCR is a biological approach to restore existing joint stability, while RSA is a prosthetic approach that mechanically replaces the shoulder by reversing joint function.

The purpose of this review is to compare SCR and RSA in the management of irreparable RCTs. This review will examine and compare the clinical effectiveness of the repair with respect to pain, range of motion, and function. There will be an emphasis on differences in biomechanical outcomes related to the restoration of shoulder stability and motion. The complications and revision rates of both repairs will also be examined to determine surgical risks and long-term durability. By comparing techniques, this review aims to guide clinical decision-making based on patient-specific factors, such as age, activity level, and surgical risk.

## Review

Search strategy

A literature search was conducted using the Preferred Reporting Items for Systematic Reviews and Meta-Analyses (PRISMA) guidelines to find studies comparing SCR to RSA in the management of irreparable rotator cuff tears (IRCTs). PubMed, Google Scholar, and Cochrane Library databases were used to search for studies published between January 2010 and the present, restricted to English-language and human clinical studies. Some resources were done through individual manual searches, specifically some meta-analyses (e.g., Werthel et al. [6], Sevivas et al. [7], Altintas et al. [9], and Sommer et al. [10]). Some narrative reviews (e.g., Di Benedetto et al. [4] and Tytgat et al. [5]) and clinical technique papers (e.g., Mihata et al. [11] and Rosales-Varo et al. [12]) were screened because of their relevance for comparative analysis.

The definitive search string used for PubMed and adapted for other databased were as follows: (“Superior Capsular Reconstruction” OR “SCR”) AND (“Reverse Shoulder Arthroplasty” OR “RSA” or “Reverse Total Shoulder Arthroplasty”) AND (“Irreparable Rotator Cuff Tear” OR “Massive Rotator Cuff Tear” OR “miRCT”) AND (“Clinical Outcomes” OR “Functional Outcomes” OR “Pain” OR “Range of Motion”).

Any duplicates in results were removed through manual screening, followed by full-text review for inclusion eligibility. The definitive list of works incorporated included those in SCR (e.g., Mihata et al. [11], Kim et al. [13], and Altintas et al. [9]) and RSA (e.g., Jarrett et al. [14], Galvin et al. [15], De La Selle et al. [16], and Barco et al. [17]), and direct comparative studies (e.g., Reddy et al. [18] and Kendirci et al. [19]) and cost analysis (e.g., Marigi et al. [20]).

Inclusion and exclusion criteria

The studies included were based on whether they directly compared SCR and RSA or had any clinical outcome data regarding either one procedure that could be used in a comparative manner. Retrospective and prospective study designs were included, along with systematic reviews that met the inclusion criteria (Tables 1, 2).

**Table 1:** Inclusion criteria. VAS: visual analog scale; ASES: American Shoulder and Elbow Surgeons Standardized Shoulder Assessment Form; UCLA: University of California, Los Angeles; RCT: randomized controlled trial.

Criterion	Description
Population	Adults (>18 years) diagnosed with massive or irreparable rotator cuff tears confirmed by imaging and/or intraoperative findings.
Intervention	Arthroscopic or open superior capsular reconstruction (SCR) using autograft or allograft materials.
Comparator	Reverse shoulder arthroplasty (RSA) performed for the same indication (irreparable rotator cuff tears or cuff tear arthropathy).
Outcomes	Studies reporting clinical, functional, or patient-reported outcomes, including pain scores (VAS), range of motion (ROM), Constant-Murley score, ASES, or UCLA Shoulder Score.
Study type	Comparative clinical studies (prospective, retrospective cohort, or RCT) or systematic reviews/meta-analyses with extractable clinical data.
Sample size	Minimum of 10 patients per group, consistent with prior meta-analytic thresholds.

**Table 2:** Exclusion criteria. SCR: superior capsular reconstruction; RSA: reverse shoulder arthroplasty.

Criterion	Description
Study type	Case reports, editorials, conference abstracts, technical notes without outcome data, and biomechanical or cadaveric-only studies.
Population	Non-human (animal or cadaveric) studies or studies involving non-adult populations.
Intervention	Studies evaluating other procedures (e.g., partial repair, tendon transfers, debridement) without SCR or RSA comparison.
Outcome reporting	Studies not reporting quantifiable clinical or functional outcomes or lacking sufficient statistical data for synthesis.

**Figure 3:**
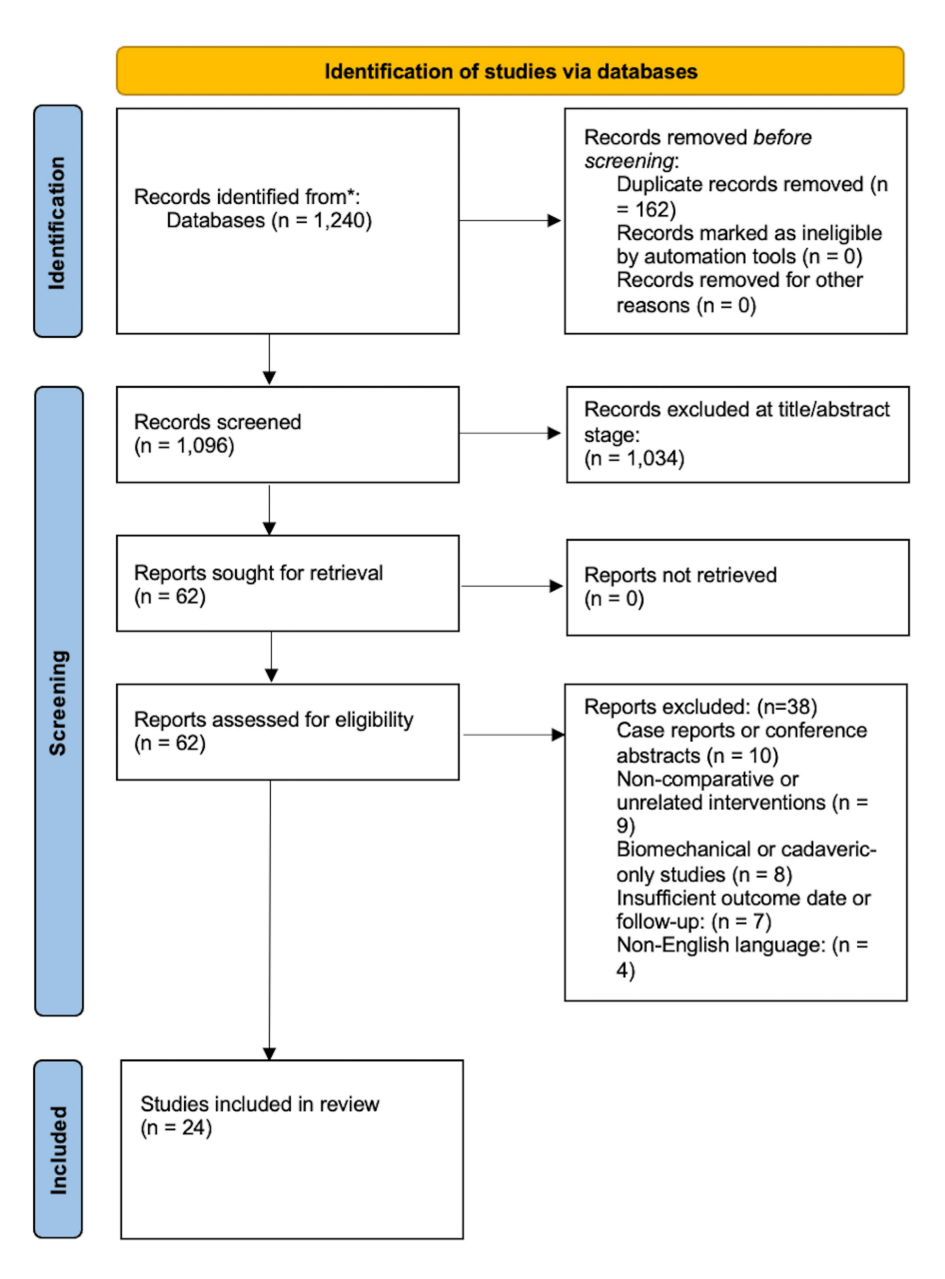
Chart detailing article selection using the PRISMA standards. PRISMA: Preferred Reporting Items for Systematic Reviews and Meta-Analyses.

The initial database search led to approximately 1,240 articles, which were reduced by removing duplicates and screening abstracts and titles. There were then 62 full-text articles left, 24 of which were used because of their comparative research and quality of data provided (Figure 3).

Superior capsule reconstruction (SCR)

Overview of SCR

SCR is an arthroscopic technique designed to address massive, irreparable posterosuperior RCTs by restoring superior glenohumeral stability. Initially described by Mihata et al. in 2012, the procedure utilizes a bridging graft to re-establish the superior capsule between the glenoid and the greater tuberosity, thereby preventing superior migration of the humeral head [11]. Over time, refinements in graft materials and fixation strategies have improved reproducibility and outcomes [6].

Laskovski et al. outlined a technical description of a simplified SCR procedure in a reproducible arthroscopic approach utilizing a nonirradiated human acellular dermal allograft [21]. The patient is positioned in the lateral decubitus position under general and regional anesthesia, with the operative arm suspended in 30° of abduction and 20° of forward flexion, under 10-15 pounds of traction. After diagnostic arthroscopy and management of any concurrent biceps or subscapularis pathology, the superior glenoid neck and greater tuberosity are debrided to expose bleeding bone, optimizing graft incorporation while preserving cortical integrity.

Two double-loaded 1.8 mm all-suture anchors are placed in the superior glenoid, one anteriorly and one posteriorly, under arthroscopic visualization [21]. An arthroscopic ruler is used to measure the graft dimensions based on glenoid-to-humerus distances, and the dermal allograft is trimmed into a trapezoid accordingly. With a minimum thickness of 3.5 mm, the graft is marked to ensure correct orientation (dermal side against bone) and introduced through an 8.5 mm cannula using a pulley technique. Medial sutures are passed through the graft and tied arthroscopically to secure it to the glenoid. Lateral fixation is achieved using triple-loaded all-suture anchors in the greater tuberosity, with sutures passed 3-4 mm from the graft edge and tied sequentially to achieve the desired contour and tension. Side-to-side sutures are used to incorporate any viable rotator cuff tissue, further stabilizing the construct.

Werthel et al., in a meta-analysis of 18 clinical studies involving 637 shoulders, highlighted the considerable heterogeneity in SCR techniques and materials [6]. Graft thickness varied from 1.5 mm (porcine xenografts) to over 6 mm (fascia lata autografts), with thicker grafts likely providing greater biomechanical resistance to superior translation. While dermal allografts were associated with lower morbidity and acceptable clinical outcomes, their thinner structure may contribute to greater variability in graft healing, emphasizing the importance of surgical precision and graft selection [6]. The main grafts used in earlier studies are fascia lata autografts taken from the patient’s thigh, which showed promising results; however, harvesting this kind of graft increases operative time and can cause morbidity at the site where the graft was taken. As a result, allografts started to be used because they are derived from acellular dermal matrices. In a systematic review by Kim et al., both grafts were effective for pain relief and functional improvement in SCR; however, fascia lata autografts showed better healing and biomechanical results. Although dermal allografts still serve as a less invasive and technically simpler graft, they have more graft failure [13].

Clinical Outcomes of SCR

Prospective outcome studies have demonstrated improvements in pain, range of motion (ROM), and function in patients with irreparable rotator cuff injuries, providing increasing evidence of SCR's clinical benefits. Note that surgical technique and graft integrity are significantly related to these results. In a prospective cohort study, Mihata et al. assessed 100 consecutive patients receiving arthroscopic SCR using fascia lata autografts, with follow-up extending up to eight years [11]. Between 2007 and 2014, 107 patients underwent SCR; seven were excluded due to unrelated medical issues. The remaining cohort included diverse tear patterns: 56 had combined supraspinatus and infraspinatus tears, 39 had additional involvement of the subscapularis, and five had involvement of the four-tendon complex, including the teres minor.

All patients underwent standardized pre- and postoperative assessments at three, six, and 12 months, as well as annually thereafter, using physical examinations, radiographs, and MRI scans. The mean American Shoulder and Elbow Surgeons Standardized Shoulder Assessment Form (ASES) score improved significantly from 31.6 to 93.3, and the Japanese Orthopaedic Association (JOA) score increased from 51.6 to 92.2 (P < 0.00001). These improvements were sustained over time and associated with significant gains in active forward elevation, particularly among patients with intact grafts. For example, patients with healed grafts achieved a mean forward elevation of 154.8°, compared to 115.0° in those with graft failure (P < 0.001). Additionally, return to pre-injury activities was high: 94% of previously employed patients resumed work, and all recreational athletes returned to sport without limitation. These results underscore the functional efficacy of SCR in the setting of irreparable rotator cuff pathology. The high healing rate and significant improvements in patient-reported outcomes support its utility in active populations, with graft integrity emerging as a critical determinant of success.

This study contributes compelling evidence that SCR not only restores shoulder biomechanics but also facilitates meaningful return to prior activity levels, making it a viable surgical strategy for patients with advanced rotator cuff degeneration. However, the study's inclusion of patients with heterogeneous RCT patterns, ranging from two-tendon to four-tendon involvement, may confound the results by combining individuals with varying prognoses. Furthermore, the variable follow-up duration, ranging from 12 to 96 months, introduces potential bias in assessing long-term graft durability and clinical outcomes, as a significant portion of the cohort (17%) was followed for less than five years. This variability in both tear complexity and follow-up time reduces the precision of long-term outcome predictions and may lead to an underestimation of late complications.

A pilot study by Rosales-Varo et al. evaluated a novel SCR technique using autologous hamstring tendons in eight patients with symptomatic IRCTs. Tendon irreparability was defined intraoperatively, supported by preoperative indicators such as acromiohumeral (AH) distance <7 mm, Patte stage 3 retraction, Goutallier grade 3-4 fatty infiltration, and Thomazeau grade 3 muscle atrophy. All surgeries were performed by a single surgeon under general anesthesia with patients in a beach-chair position. Following debridement, the graft was anchored medially at the 12 o’clock position of the glenoid and laterally in the humeral head at two fixation points: anterosuperiorly near the bicipital groove and posterosuperiorly near the infraspinatus footprint. Standardized postoperative rehabilitation included immobilization, followed by staged active and strengthening exercises [12].

At the one-year follow-up, the Constant score improved from 49 to 77.25 (P < 0.01), and forward flexion increased from 99.3° to 142.5° (P < 0.01). Importantly, the acromiohumeral distance increased from 5.25 mm to 8.18 mm, suggesting a biomechanical restoration of superior joint stability. No graft failures or reoperations were reported during the follow-up period. These results suggest that autologous hamstring graft SCR can significantly improve function and joint stability in RCT-induced pseudoparesis of the shoulder cases, even in the presence of advanced fatty degeneration. Furthermore, this study is limited by its small sample size (n = 8), which reduces statistical power and limits the generalizability of the findings. Additionally, the absence of a control group prevents any definitive conclusions about the effectiveness of the intervention compared to other treatments. The 12-month follow-up period is too short to evaluate long-term outcomes or potential graft failures. Finally, being a single-surgeon, single-center study, the results may reflect individual expertise rather than a broadly applicable technique.

These studies collectively support the primary advantages of SCR, which include long-lasting gains in shoulder ROM and scores, biomechanical stabilization of the joint (as evidenced by the restoration of acromiohumeral distance), and the ability to resume everyday and athletic activities. Both studies, however, demonstrate that these outcomes are closely related to graft healing, underscoring the importance of a meticulous surgical approach and suitable patient selection [11,12].

Limitations of SCR

Despite promising short-term results, SCR carries several limitations that warrant careful consideration. These include high variability in graft integrity, frequent complications requiring reoperation, and limited long-term data, particularly in younger and active patients.

Altintas et al., in a systematic review of seven clinical studies involving 344 shoulders, reported an overall graft retear rate of 13%, with most retears occurring at the humeral attachment site [9]. Of the total cohort, 30 patients (nearly 9%) required secondary procedures following SCR, including 15 revisions, seven conversions to RSA, and four infection-related debridements. While patient satisfaction was high (72.9%-90% across studies), the structural integrity of the graft was a key determinant of clinical success. This review also highlighted that most of the included studies were rated as fair to low in quality using the Modified Coleman Methodology Score, which also identified methodological issues. The lack of long-term follow-up (mean duration: 15-48 months) and inconsistent reporting of outcome measures further limit the generalizability of the findings.

In a separate systematic review of 14 studies encompassing 507 shoulders, Sommer et al. found graft failure rates ranging from 8% to 70%, with particularly high failure rates associated with dermal allografts compared to fascia lata autografts [14]. When reoperations were included, overall complication rates reached up to 36%. Notably, many follow-up operations were performed due to mechanical failures, such as graft rupture, anchor pullout, or loss of fixation; others addressed persistent pain, stiffness, or functional deficits. Sommer et al. also demonstrated that donor-site morbidity, prolonged surgical time, and biologic graft incorporation remain unresolved concerns in autograft-based reconstructions [10].

Another challenge is the variability in surgical technique. Across the studies included in both reviews, anchor types, numbers, graft thicknesses, and fixation angles varied substantially, impeding reproducibility and comparative analysis. In some instances, failure may be due not to the graft material itself but to improper tensioning or suboptimal placement. Furthermore, both studies emphasized that SCR did not stop the progression of the disease, specifically in individuals with extensive fatty infiltration or pseudoparalysis, which raises questions about its long-term usefulness in complex cases [9].

While SCR remains a promising joint-preserving procedure, particularly in younger patients with preserved glenohumeral cartilage, its limitations, including graft retear, the need for revision surgery, and the heterogeneity of technique, must be acknowledged. To determine the long-term durability and function of SCR within the larger therapy strategy for irreparable rotator cuff injuries, high-quality comparative trials with long-term follow-up are still needed [9,10].

Reverse shoulder arthroplasty (RSA)

Overview of RSA

RSA is a revolutionary surgical procedure that has gained widespread popularity due to its promising results in repairing RCTs. It is meant for patients with massive RCT without arthritis after all other conservative measures, like slinging and tendon repair, have failed.

The technique reverses the natural ball-and-socket anatomy and alters the shoulder biomechanics. It places a prosthetic ball on the glenoid that will be inserted into a socket on the proximal humerus. Subsequently, the center of rotation of the glenohumeral joint will be stabilized, and the deltoid muscle will compensate for deficiencies in the rotator cuff. This biomechanical alteration allows for improvement in arm elevation and shoulder function in the absence of a functional rotator cuff.

To perform the surgery, patients will be put under anesthesia, and the surgeon will access the glenohumeral joint. First, a metal-backed baseplate will be secured directly onto the glenoid using screws. Next, a metal glenosphere will be attached to the baseplate to act as the new articulating ball. Finally, a humeral socket will be placed onto the proximal humeral stem and will work as the opposing articulating surface of the glenosphere. Together, all three parts make the new ball-and-socket for the shoulder with a more medial and distal center of rotation. The deltoid is then allowed to provide more assistance to the joint for arm movement [14].

Clinical Outcomes of RSA

A systematic review and meta-analysis by Galvin et al. (2022) evaluated the outcomes of reverse total arthroplasty based on 52 studies published between 2005 and 2020. The data covered a total of 5,824 shoulders with a median age at surgery of 71.9 years and a minimum follow-up of two years. Patients had a mean improvement of 56° in active flexion, 50° in active abduction, and 14° in active external rotation. In addition, the study showed significant improvement in patients' Constant score (a test used to assess shoulder function) and the ASES [15]. Limitations of the study include incomplete data from the studies the researchers used. Many outcomes and demographics could not be studied.

In another study by De La Selle et al. (2023), researchers looked at 79 patients who underwent RSA for massive RCT or cuff tear arthropathies (CTAs) between 2011 and 2013 and evaluated the long-term effects of the surgery. Patients had a minimum follow-up of 7.4 years and were evaluated based on Constant scores and the ASES. The study assesses the Constant score and ASES outcome of patients who had RSA for massive RCT versus CTAs, with no significant difference between the two. However, when comparing the pre- versus postoperative Constant scores and ASES, patients show a significantly higher score after undergoing an RSA, with a mean follow-up of 8.9 years [16]. Limitations of this study include the small sample size of 79, which would not allow for the study of cofounding factors.

Together, these studies affirm the value of RSA and emphasize the benefit of the procedure for RCTs that do not heal with conservative measures. By changing the biomechanics of the shoulder joint to allow for deltoid compensation, patients gain short-term and long-term benefits after, rather than before, the surgery. It increases their shoulder ROM, Constant score, and ASES.

Limitations of RSA

Although RSA yields significant improvements in pain relief and shoulder function, it is not without complications that must be carefully considered during surgical planning and patient counseling. The most common types of complications include instability, infection, notching, loosening, nerve injury, acromial and scapular fractures, intraoperative fractures, and component disengagement [18]. Instability usually occurs within the first six months after the procedure is done, and the major concern is dislocation.

The mechanism of RSA relies heavily on the deltoid muscle to compensate for raising the arm, and instability can occur with failure to achieve the correct tension of the deltoid. In a study by Markes et al. (2020), stability can be restored by maximizing deltoid and soft tissue tension. To do this while avoiding impingement, the researchers used three methods: (1) lateralizing and/or upsizing the glenosphere to an inferior position on the glenoid; (2) use of a more constrained polyethylene insert; (3) distalizing the humerus by increasing the polyethylene thickness and/or the thickness of the humeral tray [22].

Another complication due to deltoid tension is the risk of fracture on a weakened acromion. Infection is a common complication of all surgeries. However, when compared to anatomic shoulder arthroplasty, RSA is reported to have higher rates of infection. The reason is not clear, but it can possibly be attributed to the increased implant surface and larger dead space [18]. When an infection is present, the most common steps in management include the complete removal of the implant and placement of an antibiotic spacer, along with the administration of IV antibiotics [22]. Another complication of RSA is an injury to the axillary nerve, which is due to its anatomical location and the nature of the procedure. Accessing the glenohumeral joint using the deltopectoral approach can lower the risk of damage.

Comparative analysis of SCR and RSA

Overview of Studies

In one retrospective cohort study, RSA and SCR were compared in patients with pseudoparesis secondary to massive irreparable rotator cuff tears (miRCTs) [18]. Patients were treated at a single institution by a fellowship-trained shoulder surgeon between 2016 and 2022. Inclusion required active forward elevation (AFE) between 45°-90°, full passive ROM, an age range of 40-70 years, symptoms for ≥6 months, and ≥12-month follow-up. Pseudoparesis was confirmed by physical examination and lidocaine injection to exclude pain inhibition. Patients with traumatic tears, prior surgery, or limited passive ROM were excluded. Out of 91 patients initially reviewed, 50 met the study’s criteria, 27 received RSA, and 23 underwent SCR. Both groups had similar starting characteristics, although RSA patients had slightly more severe arthritis.

In a comparative cohort study conducted at a tertiary referral center for shoulder disorders, the clinical efficacy of arthroscopic superior capsular reconstruction (aSCR) using tensor fascia lata (TFL) autograft was evaluated against RSA in patients with miRCTs and early-stage cuff tear arthropathy (Hamada grade 1-2). The cohorts consisted of 20 patients each, with mean ages of 61.85 ± 7.56 years for the aSCR group and 71.10 ± 6.42 years for the RSA group (P = 0.004). Follow-up durations differed significantly: 22.3 ± 8.4 months for aSCR and 32.5 ± 8.1 months for RSA (P = 0.007). Despite age and follow-up discrepancies, both groups were otherwise demographically comparable. Surgical techniques were standardized and performed by a single senior surgeon. The aSCR involved harvest and three-layer folding of a TFL autograft with fixation via suture anchors in the glenoid and humeral head, following Mihata’s technique. RSA procedures utilized uncemented components with glenosphere implantation [19].

Clinical Outcomes

In the retrospective study by Reddy et al., before surgery, the shoulder elevation was nearly the same in both groups (RSA: 65°; SCR: 67°). After surgery, RSA patients showed a greater improvement in elevation (by 89° on average) compared to SCR patients (73° improvement). However, the final arm elevation reached was similar (RSA: 154°; SCR: 140°), and this difference was not statistically significant. Almost all patients regained the ability to lift their arm, with 96% in the RSA group and 91% in the SCR group. Internal rotation, the ability to rotate the arm inward, was better preserved in the SCR group, scoring 6.9 points versus 4.6 in the RSA group on a standardized scale. External rotation was similar between groups. When it came to patient-reported outcomes, those who had RSA reported better overall shoulder function (average score: 91% vs. 69%) and less pain (average pain score: 0.6 vs. 2.2). Both groups had similar scores on a common shoulder assessment scale (ASES). Each group had one reoperation, one due to RSA dislocation and the other due to SCR failure, which required conversion to RSA [18]. After adjusting for differences such as age and arthritis severity, the data still showed that RSA was associated with higher satisfaction and lower pain. At the same time, SCR continued to demonstrate better internal rotation.

In the comparative cohort study by Kendirci et al., both interventions yielded statistically significant postoperative improvements across all parameters (P < 0.05). Between-group comparison showed no significant difference in most outcome deltas, except for shoulder abduction, which was superior in the RSA group (P = 0.003). At final follow-up, the mean ASES score improved to 87.9 ± 9.3 in RSA patients versus 80.5 ± 13.4 in the aSCR cohort. Visual analog scale (VAS) scores were low in both groups (RSA: 1.2 ± 0.7; aSCR: 1.3 ± 0.9). In the aSCR group, MRI demonstrated graft integrity in 13 of 20 patients (65%), with no significant clinical differences between graft-healed and graft-failure subgroups [19]. No complications were observed in the RSA cohort. In the aSCR group, two minor complications occurred: one donor site hematoma and one case of acromioclavicular arthritis.

Complications and Limitations

The findings in the study by Reddy et al. suggest that both RSA and SCR are highly effective for reversing pseudoparesis in miRCTs, with resolution rates above 90%. RSA yielded greater subjective function and pain relief, possibly due to prosthetic biomechanics and patient selection favoring lower activity levels. However, RSA was associated with inferior internal rotation (IR) recovery, a common limitation likely attributable to implant constraints and surgical technique. These data underscore the need for individualized treatment selection: RSA may be preferable in patients prioritizing pain relief and global shoulder function, whereas SCR may benefit those requiring greater IR or desiring joint preservation. Further prospective studies are warranted to refine surgical indications and to compare long-term outcomes [18].

A limitation of this study includes the non-randomized allocation of patients to treatment groups, which introduces selection bias and potential confounding by indication. The small sample size further limits the statistical power to detect meaningful differences between groups. Additionally, the study was conducted at a single center by one experienced surgeon, which restricts the generalizability of the findings. Lastly, the relatively short follow-up period of 12 months may not adequately capture long-term outcomes or complications, particularly those relevant to implant durability and functional longevity.

The study by Kendirci et al. has promising results that support aSCR with TFL autograft as a viable biological alternative to RSA for selected miRCT patients with early arthropathy, with comparable short-term outcomes and the added benefit of joint preservation. However, graft integrity remains a key variable influencing long-term success, and additional prospective studies with longer follow-up and matched cohorts are warranted to establish optimal indications and durability of this approach [19].

Additionally, some limitations of this study were that the study groups were not matched for key variables such as age and follow-up duration, with the RSA cohort being significantly older and having a longer mean follow-up, which may confound comparative outcome analyses. Additionally, the relatively small sample size in each group limits the statistical power to detect subtle differences or rare complications. The single-surgeon, single-center design further restricts the generalizability of the findings across different clinical settings and surgical expertise. Moreover, the short-term follow-up period precludes assessment of long-term outcomes, including graft durability in the aSCR group and implant survivorship in the RSA cohort.

Rehabilitation

In a systematic review by Lavin et al., the rehabilitation protocols of SCR for irreparable rotator cuff repair were investigated and showed great variability in the timing and level of postoperative management. In rehabilitation, there was an emphasis on gradual mobilization and protecting the graft, given that SCR involves the usage of a biological graft to regain shoulder stability. The usage of an abduction brace for a period of immobilization ranging from three to six weeks, as well as full immobilization, was often recommended to prevent tension on the graft. In some studies within the systematic review, passive range of motion (PROM) was initiated after six weeks postoperatively, while others were prolonged with fears of negatively affecting the graft. Active assisted range of motion (AAROM) and active range of motion (AROM) were initiated six to eight weeks postoperatively, and strengthening and higher-intensity activities were prolonged to three to 12 months postoperatively, depending solely on patient progress. In general, rehabilitation of SCR for IRCTs involves a cautious approach to promote tissue/graft integration and minimize superior humeral head migration before strength restoration [23].

In a systematic review by Lu et al., the rehabilitation protocols of RSA for irreparable rotator cuff repair were investigated and showed greater consistency in protocols, and focused on prosthetic integration with tissues. Like in SCR rehab, RSA requires generally four to six weeks of immobilization to allow for tissue healing and deltoid adaptation to the prosthetic. However, PROM is often recommended during the latter half of this period to maintain joint mobility. At six to 12 weeks postoperatively, AAROM and AROM are introduced, and strengthening exercises targeting the deltoid and periscapular muscles are started at eight to 12 weeks. The strengthening exercises are started earlier in RSA compared to SCR because aggressive and function-oriented rehabilitation is important to help with altered joint mechanics and deltoid compensation because of the prosthesis. Furthermore, there is an emphasis on restoring the ROM and functionality to incorporate the prosthesis while avoiding instability and component loosening [24].

Comparatively, SCR rehabilitation is protective and prolonged to emphasize graft preservation, resulting in a postoperative recovery that ranges from six to 12 months. Meanwhile, RSA rehabilitation is focused on regaining functioning to incorporate a prosthesis into neighboring tissues through mechanical usage and strengthening, which takes a shorter time, at around three to six months. The difference in the protocol and timelines reflects the main components of either procedure in repairing IRCTs, given that one relies on incorporating a graft (SCR) and the other on incorporating a prosthesis (RSA) [23,24].

Cost Comparison

A study was conducted at a high-volume tertiary referral center to evaluate the short-term outcomes and costs of two surgical treatments for IRCTs (SCR and RSA) [20]. The analysis included 108 shoulders treated between 2018 and 2020, with 20 undergoing SCR and 88 receiving RSA. Patients were selected based on advanced tendon degeneration confirmed through imaging and intraoperative findings. Key exclusions included patients with severe arthritis, neurologic impairment, or additional unrelated procedures. Both procedures followed standardized perioperative protocols, with surgeons selecting implants and grafts based on clinical preference. SCR involved the use of a dermal allograft and a greater number of suture anchors to secure the graft, while RSA used standard prosthetic implants. The average patient receiving RSA was nearly a decade older than those undergoing SCR, and SCR was more often performed in male patients [20].

The surgical duration for SCR was substantially longer, over twice that of RSA. This extended operating time, in combination with more expensive graft materials and increased anchor use, drove up SCR costs. On average, SCR required about eight suture anchors per procedure, compared to around four in RSA. Additionally, the dermal allograft used in SCR was approximately 3.5 times more expensive than graft materials typically used in RSA. Cost analysis revealed that SCR was the most expensive option across all phases of care. Total standardized costs, including preoperative imaging, the surgical procedure, and 90 days of postoperative care, averaged around $20,800 for SCR, compared to about $17,200 for RSA. The surgical episode alone (hospitalization and operative costs) was roughly $3,600 more for SCR. Implant and operating room costs were the most significant contributors in both procedures, but the use of consumables and surgical time was notably higher for SCR.

Despite these cost differences, short-term outcomes over 90 days were comparable [24]. There were no reoperations in either group, and complications were rare, affecting just a few patients across both cohorts. The most common issues were minor and included wound problems and postoperative pain. Only one patient, in the RSA group, required readmission due to a medical issue unrelated to the shoulder surgery. This study provides a direct, real-world cost comparison between SCR and RSA within a standardized healthcare system. While both procedures demonstrated similar safety in the short term, SCR incurred significantly higher costs without a corresponding reduction in complications or improved early outcomes. These findings suggest that in the early postoperative period, RSA may offer greater economic value, particularly when cost containment is a priority. However, longer-term outcome data are needed to assess the cost-effectiveness of each approach fully [20].

Summary

Statistical Summary

The statistical synthesis of this review focused on outcomes in ROM, pain scores, and complication rates between SCR and RSA. Eight of the 24 studies contained quantitative data that were extractable and useful for pooled comparison or a structured summary. The quantitative data were extracted as standard deviations and means for continuous variables, and event counts for binary outcomes. The direct statistical comparisons possible (e.g., Reddy et al. [18] and Kendirci et al. [19]) had mean differences that were standardized using standard mean deviation and expressed with a 95% confidence interval. There was heterogeneity in the study designs, with prospective comparative studies (e.g., Reddy et al. [18] and Kendirci et al. [19]) all the way to meta-analyses (e.g., Werthel et al. [6], Sevivas et al. [7], and Galvin et al. [15]). Therefore, a random effects model was used for outcomes reported by ≥3 comparable studies. On the other hand, heterogeneity was measured using the I^2^ statistical analysis, with an I^2^ greater than 50% showing significant heterogeneity. For instance, Werthel et al. [6] and Altintas et al. [9] had pooled outcomes in Constant and ASES scores with high interstudy heterogeneity at 68-74% in SCR. In Sevivas et al. [7] and Galvin et al. [15], data were pooled for RSA because of significant variance in the variables of age and preoperative function. In areas where there was overlap between patient populations, data were cross-checked to prevent any double counting (Table 3).

**Table 3:** Studies mentioned within statistical analysis and their measures. Studies included in the statistical analysis and corresponding outcome measures. SCR: superior capsular reconstruction; RSA: reverse shoulder arthroplasty; LTT: lower trapezius transfer; ROM: range of motion; ASES: American Shoulder and Elbow Surgeons Standardized Shoulder Assessment Form; VAS: visual analog scale; SMD: standardized mean difference; CI: confidence interval; ANOVA: analysis of variance; I²: I-squared statistic (measure of heterogeneity); RCT: randomized controlled trial; N/A: not applicable.

Study	Design	Intervention	Comparison	Outcomes analyzed	Statistical method used	Reported heterogeneity	Included pooled comparison
Werthel et al. [6]	Systematic review & meta-analysis	SCR	—	Constant, ASES, ROM	Random effects (SMD, 95% CI)	I² = 68–74%	Y (SCR pooled)
Sevivas et al. [7]	Systematic review & meta-regression	RSA	—	Constant, ASES, VAS	Random-effects, meta-regression	I² = 61%	Y (RSA pooled)
Altintas et al. [9]	Systematic review	SCR	—	Constant, ASES	Random effects	Not reported	Y (SCR pooled)
Sommer et al. [10]	Systematic review	SCR	—	Complications	Descriptive frequencies	N/A	Y (Complication rates)
Galvin et al. [15]	Systematic review & meta-analysis	RSA	—	Constant, VAS, complications	Random effects	I² = 55%	Y (RSA pooled)
Reddy et al. [18]	Comparative cohort	SCR	RSA	Constant, ASES, VAS, ROM	Independent t-test; SMD (computed)	Not reported	Y (Direct comparison)
Kendirci et al. [19]	Comparative cohort	SCR	RSA	Constant, ROM, complications	Mann–Whitney U test	N/A	Y (Direct comparison)
Marigi et al. [20]	Comparative cost/outcome study	SCR, RSA, LTT	RSA	Cost, complication rate	Descriptive, ANOVA	N/A	Partial (nonclinical outcomes)
Mihata et al. [11]	Case series	SCR (autograft)	—	ROM, strength	Paired t-test	N/A	X
Rosales-Varo et al. [12]	Technical & clinical report	SCR (hamstring autograft)	—	Pain, ROM	Descriptive	N/A	X
Lavin et al. [23]	Systematic review	SCR	—	Rehab protocols	Narrative	N/A	X
Lu et al. [24]	Systematic review	RSA	—	Rehab outcomes	Narrative	N/A	X

Quantitative pooling was not possible in studies that lacked consistent reporting units (e.g., Rosales-Varo et al. [12]) and those with mixed interventions (e.g., Marigi et al. [20]); therefore, data were summarized in a qualitative manner that emphasized the magnitude of improvement rather than focusing on significance testing. The subgroup analyses were graded conceptually using the following factors: (1) age: younger patients (<65 years) favor SCR vs. older patients (>70 years) favor RSA [7,9,15,18,19]; (2) graft type: autograft vs. dermal allograft in SCR [9,11-13]; (3) follow-up duration: short-term outcomes (<24 months) vs. long-term outcomes (>24 months) [6,15,16,18]; (4) rehabilitation protocol: early AROM/AAROM initiation vs. late AROM/AAROM initiation [23,24].

Where data were available, publication bias was looked at in meta-analytic studies using Egger’s regression test. However, because of limited direct comparative studies, there was no independent pooled meta-analysis in this review. Furthermore, the findings are shown in a narrative manner using quantitative evidence provided.

Discussion

RSA and SCR are both established surgical options for treating IRCTs, especially in cases complicated by pseudoparesis or cuff tear arthropathy (CTA). While both techniques have shown promising results, they each have distinct advantages, limitations, and long-term implications that must be carefully considered when determining the most appropriate surgical intervention for a given patient.

A systematic review by Galvin et al. (2022) highlighted the positive outcomes of RSA, with substantial improvements in shoulder function, including a mean increase of 56° in active flexion, 50° in abduction, and 14° in external rotation across 5824 shoulders [15]. Patients also reported significant gains in functional scores such as the Constant score and the ASES [15]. These outcomes are supported by De La Selle et al. (2023), whose long-term follow-up of patients treated with RSA for massive RCTs or CTA found significant postoperative improvements in both the Constant score and ASES, with no significant difference between the two subgroups [16]. These results underscore RSA’s effectiveness in improving shoulder motion and function, especially in older populations or those with advanced arthritis or irreparable tears.

However, RSA is not without risks, including the potential for complications like instability, infection, nerve injury, and fractures. The mechanism of instability, which often occurs in the first six months postoperatively, is related to the deltoid’s compensatory role in arm elevation. Successful RSA relies on achieving optimal tension in the deltoid, which can be challenging and, if not done correctly, can lead to poor outcomes. Infection rates in RSA are also higher compared to anatomical shoulder arthroplasty, likely due to the larger implant surface area and dead space created by the procedure. Nerve injury, especially to the axillary nerve, remains a concern, though the deltopectoral approach may reduce this risk [17,22].

In contrast, SCR provides a joint-preserving alternative to RSA, particularly for younger and more active patients. A comparative study of RSA and SCR found that while both procedures effectively addressed pseudoparesis in patients with miRCTs, RSA provided superior pain relief and overall shoulder function, with a higher patient-reported satisfaction rate. However, SCR preserved better internal rotation, making it a preferable option for patients who place a high value on this aspect of shoulder motion [18].

SCR, typically performed arthroscopically, involves the use of autografts or allografts to reconstruct the superior capsule of the shoulder, thereby improving joint stability and function. However, graft failure is a significant limitation, with retear rates ranging from 8% to 70% depending on the type of graft and surgical technique used. Autograft options, such as fascia lata or hamstring tendons, tend to offer better durability compared to allografts. However, they are not without their own risks, including donor-site morbidity and longer operative times. Additionally, while SCR provides the benefit of joint preservation, it does not halt the progression of arthritis in patients with advanced stages of CTA, limiting its long-term efficacy in more complex cases (Table 4) [9,10].

**Table 4:** Comparison of SCR to RSA in parameters of importance based on data. ROM: range of motion.

Parameter	Superior capsular reconstruction (SCR)	Reverse shoulder arthroplasty (RSA)
Age of the ideal patient	40–65 years old [21]	60–85 years old [12]
Functional improvement (ROM)	Forward flexion: 120°–160° [7,9]. Abduction: 100°–140° [7,21]. Internal rotation: 40°–60° [15]	Forward flexion: 90°–130° [9,12]. Abduction: 110°–160° [9,12]. Internal rotation: 0°–20° [12]
Pain relief	70%–90% report significant pain reduction [13,21]	85%–95% report significant pain reduction [10,14]
Strength improvement (deltoid)	Moderate improvement [7,15]	Significant improvement, especially in abduction [12]
Re-tear rate (SCR)/instability (RSA)	5%–30% [13,21]	5%–15% [10,14]
Rehabilitation duration	6–12 months for full recovery [7,21]	6–9 months for full recovery [9,12]
Revision rate	10%–30% due to graft failure [13,21]	5%–10% primarily due to implant failure [10,14]

Both RSA and SCR have demonstrated significant improvements in patient outcomes, but these benefits come with their own sets of challenges. RSA tends to be more effective for patients with severe arthritis or advanced joint degeneration, as it offers pain relief and functional improvement that may not be achievable with SCR. However, RSA is associated with a higher risk of complications, including infection and instability, which require careful management during the postoperative period. SCR, on the other hand, offers a biologically based alternative that preserves the native joint and avoids the need for a prosthetic implant. This technique is particularly beneficial for younger patients or those with lower activity demands, and it has shown success in improving motion and stability, especially in patients with more preserved joint structures. However, SCR requires careful attention to graft integrity, as failure to heal properly can lead to poor functional outcomes, and it is less effective in cases where there is significant arthritis or fatty infiltration.

Recent studies also highlight the cost differences between the two procedures. In a study comparing RSA and SCR, RSA was found to be more cost-effective in the short term, primarily due to lower graft costs and shorter surgical times. SCR, particularly when using autografts or allografts, tends to incur higher costs due to longer surgical durations and more expensive materials. These cost differences, however, do not necessarily translate into superior long-term outcomes, as the durability of the graft and the progression of arthritis in SCR patients can impact the need for future interventions (Table 5) [24].

**Table 5:** Comparison of the costs associated with SCR and RSA based on the data.

Aspect	Superior capsular reconstruction (SCR)	Reverse shoulder arthroplasty (RSA)
Initial cost	$15,000–$30,000 [17,21]	$20,000–$40,000 [12,17]
Cost of materials	Graft materials: $2,000–$5,000 (autograft or allograft) [17,21]. Surgical instruments: $5,000–$10,000 (specialized tools for arthroscopy) [21]	Implants: $5,000–$10,000 (prosthetic components) [12,18]. Surgical instruments: $3,000–$7,000 (standard tools for implant placement) [12]
Revision surgery cost	Cost for re-tear or graft failure: $15,000–$25,000 (for potential revision surgeries) [13,21]	Cost for implant revision: $25,000–$40,000 (for revision of prosthetic components) [12,14]
Total lifetime cost	If graft fails: $25,000–$55,000 (including revisions and additional therapies) [13,21]	Implant failure or dislocation: $30,000–$50,000 (revision or replacement) [10,12]
Insurance coverage	Often reimbursed by insurance but may require special authorization due to complexity [21]	Typically covered by insurance, with some restrictions based on patient age and activity level [10]
Graft failure limitation	Graft failure rate: 10%–30% [13,21]	N/A

## Conclusions

In conclusion, while both RSA and SCR provide effective solutions for managing miRCTs and related shoulder dysfunctions, the choice between the two should be guided by the patient's age, activity level, comorbidities, and the severity of joint degeneration. RSA is more suited to older patients with significant arthritis or joint degeneration, providing superior pain relief and function but at the cost of a higher complication rate. SCR, on the other hand, is a viable joint-preserving option for younger, more active patients, particularly those with less severe arthritis. However, its long-term success is heavily dependent on graft integrity and the surgeon’s technical skill. Both procedures are associated with specific risks and challenges, and further high-quality, long-term studies are needed to refine patient selection criteria and improve the reproducibility of outcomes across different surgical settings.
